# Hyomental distance measured ultrasonography versus weight-based criteria for laryngeal mask size selection in children: a randomized controlled trial

**DOI:** 10.1186/s12871-025-03303-8

**Published:** 2025-08-25

**Authors:** Sherif Kamal Arafa, Mohammed Said ElSharkawy, Mostafa Mohamed Shaheen, Ahmed Shama

**Affiliations:** 1 Faculty of Medicine, Anesthesia, Surgical Intensive Care and Pain Management Department, Kafr Elsheikh University, Kafr Elsheikh, 33512 Egypt; 2 Faculty of Medicine, Anesthesiology, Surgical Intensive Care and Pain Medicine Department, Tanta University, Tanta, Egypt

**Keywords:** Laryngeal mask airway, Pediatric, Ultrasonography, Hyomental distance, Airway management

## Abstract

**Background:**

Typically, when choosing a size for a child’s laryngeal mask airway (LMA), weight is considered; however, this approach has limitations. This study evaluated the efficacy of ultrasonography (US) measured hyomental distance (HMD) compared to weight-based criteria for optimizing the selection of LMA size in pediatrics.

**Methods:**

This randomized controlled trial included 60 children aged 3–14 years scheduled for surgery requiring LMA. Patients were randomized equally to two groups. LMA size was selected based on either US-measured HMD [< 2 cm for size 2, 2–3 cm for size 2.5, and > 3 cm for size 3] in the HMD group or weight-based [size 2 for 10 to 20 kg, size 2.5 for 20 to 30 kg, and size 3 for 30 to 50 kg] in the control group (C Group).

**Results:**

The success rate (the proportion of cases where the first-selected LMA size required no adjustment or alternative devices) was significantly higher in the HMD group compared to the control group (93.33 vs. 70%, *p* = 0.041). LMA placement five-point optimization scores (1: easiest insertion, 5: impossible) and a two-point scale (0: optimal cuff symmetry/esophageal alignment, 1: suboptimal) were significantly better in the HMD group (*p* < 0.05). The HMD method demonstrated superior predictive value for successful intubation with 98% sensitivity and 72.7% specificity, compared to weight-based criteria with 87.8% sensitivity and 9.1% specificity (*p* < 0.001).

**Conclusions:**

US-measured HMD in pediatrics provides a superior method for choosing LMA size in children than traditional weight-based criteria, resulting in improved intubation success rates, better optimization scores, and higher predictive accuracy, aligning with prior studies in neonates and adults. We recommend HMD as a more reliable metric for pediatric LMA sizing.

**Trial registration:**

The Pan African Clinical Trial Registry was notified about the trial before patient recruitment (PACTR202306740935710, registration date: 09 Jun 2023).

## Background

In the field of pediatric anesthesia and emergency medicine, the laryngeal mask airway (LMA) has become an indispensable tool for managing airways, offering advantages such as ease of use, reduced airway trauma, and improved patient satisfaction compared to endotracheal intubation [1, 2]. However, selecting the appropriate LMA size is crucial for its practical and safe application, particularly in children [3].

LMA size selection has traditionally relied on weight-based criteria [4]. Nevertheless, this method has limitations, as it fails to account for individual anatomical variations and the non-linear relationship between body weight and upper airway dimensions in children [5]. Furthermore, in emergencies, a patient’s weight may be unknown or difficult to estimate accurately [6].

Researchers have explored alternative methods for LMA size selection to address these limitations. Some have proposed using anatomical measurements, such as finger width [7] or auricle size [5], as predictors of appropriate LMA size. While these approaches show promise, their applicability across diverse patient populations remains unclear [8].

Ultrasonography (US) has gained attention for perioperative airway evaluation [9, 10]. Recent studies have explored airway US measurements, including the hyomental distance (HMD), which represents the distance between the hyoid bone and the tip of the chin, and the HMD ratio (HMDR), defined as the ratio of the HMD in maximal head extension to that in the neutral position, concerning their correlation with pharyngeal cavity length for optimizing LMA size selection [11–14]. However, the accuracy of these clinical measurements varies, likely due to operator-dependent factors and difficulties in landmark identification [15].

Given the limitations of weight-based criteria and the promising results of US-guided airway assessment, this research intended to investigate the potency of US-HMD measurements in optimizing LMA size selection for pediatric patients.

## Methods

This prospective, randomized, double-blind-controlled study was conducted on 60 children aged 3–14 years with American Society of Anesthesiologists (ASA) physical status I or II scheduled for elective general pediatric surgeries under general anesthesia using an LMA (such as hernia repair, circumcision, and minor orthopedic procedures) at Kafr El Sheikh University Hospitals, Egypt, from April 2023 to October 2023.

The study was approved by Kafr El Sheikh University Ethical Committee (ID: MKSU 51-2-18) and registered on the Pan African Clinical Trial (ID: PACTR202306740935710, registration date: 09 Jun 2023). Written informed consent was obtained from all participants’ parents or legal guardians.

Exclusion criteria comprised developmental delay (potential anatomical airway abnormalities), drug hypersensitivity (hypersensitivity to medications used as propofol, atracurium, fentanyl), difficult anatomical airway (congenital anomalies as Pierre Robin sequence, Treacher Collins syndrome, Down syndrome, and craniofacial abnormalities, micrognathia, macroglossia, or airway tumors), past surgeries on the neck, infections of the upper respiratory tract (active infections with fever, purulent nasal discharge, or productive cough), congenital heart disease, or emergency surgeries (potential for aspiration).

### Randomization and blindness

In sealed opaque envelopes, participants were randomly assigned to two equal groups (*n* = 30 each) using computer-generated random numbers (https://www.randomizer.org/). In the HMD group, LMA (Classic™) size selection was based on US-measured HMD: <2 cm for size 2, 2–3 cm for size 2.5, and > 3 cm for size 3 [16]. The control group (C Group) selected LMA size according to weight-based manufacturer recommendations: size 2 is recommended for those weighing 10 to 20 kg, size 2.5 for individuals between 20 and 30 kg, and size 3 for those weighing 30 to 50 kg.

The study employed a triple-blinding design to minimize measurement, placement, and evaluation bias. The first anesthesiologist, a specialist with over five years of focused training in point-of-care US for pediatric airway applications, performed US assessments. The second anesthesiologist, with 10 years of experience in pediatric airway management, was responsible for LMA insertion and mask ventilation while remaining blinded to group allocation to reduce variability. The third anesthesiologist, an assessor with eight years of experience in pediatric airway management, was formally trained in the five-point and two-point scoring systems and evaluated outcomes without knowledge of group assignments. Additionally, patients were kept blinded throughout the trial.

Pre-anesthetic management encompassed a thorough patient history, physical assessment, and standard laboratory testing. Patients fasted 2 h for clear fluids, 4 h for unclear fluids, and 6 h for solid foods before surgery.

An intravenous line was placed at the patient’s peripheral. One hour before surgery, 0.01 mg/kg of atropine was given as premedication. Monitoring included pulse oximetry, electrocardiogram, temperature, capnogram, and noninvasive blood pressure.

We used propofol (2 mg/kg) and fentanyl (1 µg/kg) intravenously to induce general anesthesia. Atracurium 0.5 mg/kg was used for muscle relaxation. US measurements of HMD were performed in both groups. Anesthesia was maintained with 1–3% sevoflurane titrated to hemodynamic stability (heart rate and blood pressure within 20% of baseline) to achieve and maintain bispectral index (BIS) values between 40 and 60, indicating an appropriate depth of anesthesia. Fresh gas flow was standardized at 1 L/min (50% O₂/air), with adjustments made to maintain end-tidal carbon dioxide between 35 and 40 mmHg.

The ease of insertion was evaluated using a five-point scale [17] to rate the ease of inserting an LMA on a scale from 1 to 5, where 1 indicates that the LMA was inserted without difficulty and 5 indicates that it was impossible to be inserted.

### The ultrasound for HMD measurements

The HMD was assessed using a US with a high-frequency linear probe (4–18 MHz).

US measurements were conducted after anesthetic induction, with the patient fully relaxed and supine with their heads extended; a craniocaudal sagittal scan was performed with the probe positioned in the submental region of the neck. The hyoid bone and the lower end of the chin were visible on the US image as hyperechoic structures. After freezing the US image, the distance between the hyoid bone and mentum was measured directly on the screen [18].

Two standard imaging planes were obtained for LMA placement assessment: a transverse image between the hyoid bone and thyroid cartilage was obtained, where the cuff shadow should appear symmetrical on both sides of the midline, and a parasagittal image of the pharynx and larynx, where the cuff and esophagus should be on the same sagittal plane. A two-point scale [19] was used to evaluate ultrasound images: Score 0 indicated optimal placement, defined as (1) a smooth and symmetrical transverse cuff tip (indicating midline alignment without rotation), and (2) the cuff and esophagus aligned on the same sagittal plane (indicating proper depth positioning), while Score 1 indicated suboptimal placement, defined as failure to meet either or both criteria (e.g., asymmetrical cuff, misaligned cuff/esophagus). Placement grades were categorized as Grades I/II for optimized placement (Score 0) and Grade III for non-optimized placement (Score 1).

At a constant gas flow rate of 3 L/min, the expiratory valve of the circular system was closed one minute after the airway was secured to determine the oropharyngeal leak pressure (OLP). When the airway pressure achieved equilibrium, a gas leak could be detected by hearing the leak or using a stethoscope positioned directly lateral to the thyroid cartilage to detect the noise. This pressure was determined to be the OLP [20].

The primary outcome was the success rate (successful intubation defined as successful LMA placement from the first time) of the predicted LMA size without needing size adjustment or employing other devices. The secondary outcomes included injury rate (mucosal trauma, bleeding, or blood on the mask), OLP measurements, and number of insertion attempts (adjustments without complete removal counted as a single attempt, but complete removal and reinsertion constituted a new attempt).

### Sample size calculation

The sample size calculation was conducted using G*Power 3.1.9.2 (Universität Kiel, Germany). A pilot study was conducted with ten cases in each group, revealing that effective intubation (the primary result) occurred in 100% of group HMD and 70% of group C. The sample size was determined using a 95% confidence interval, 90% statistical power, a group ratio of 1:1, and the inclusion of two additional cases in each group to mitigate dropout rates. Consequently, we enlisted 30 patients in each cohort.

### Statistical analysis

The statistical analysis was performed utilizing SPSS v27 (IBM^©^, Armonk, NY, USA). The normality of the data distribution was evaluated utilizing the Shapiro-Wilk test and histograms. The quantitative parametric data were presented as the mean and standard deviation (SD) and analyzed using an unpaired Student’s t-test. The quantitative non-parametric data were represented as the median and interquartile range (IQR) and analyzed using the Mann-Whitney test. The qualitative variables were presented as frequency and percentage (%) and examined with the Chi-square or Fisher’s exact test. The sensitivity, specificity, positive predictive value (PPV), negative predictive value (NPV), and accuracy for head and neck morphology (HMD) and weight-based parameters in predicting effective intubation were computed. A two-tailed P value below 0.05 was considered statistically significant.

## Results

**Fig. 1:**
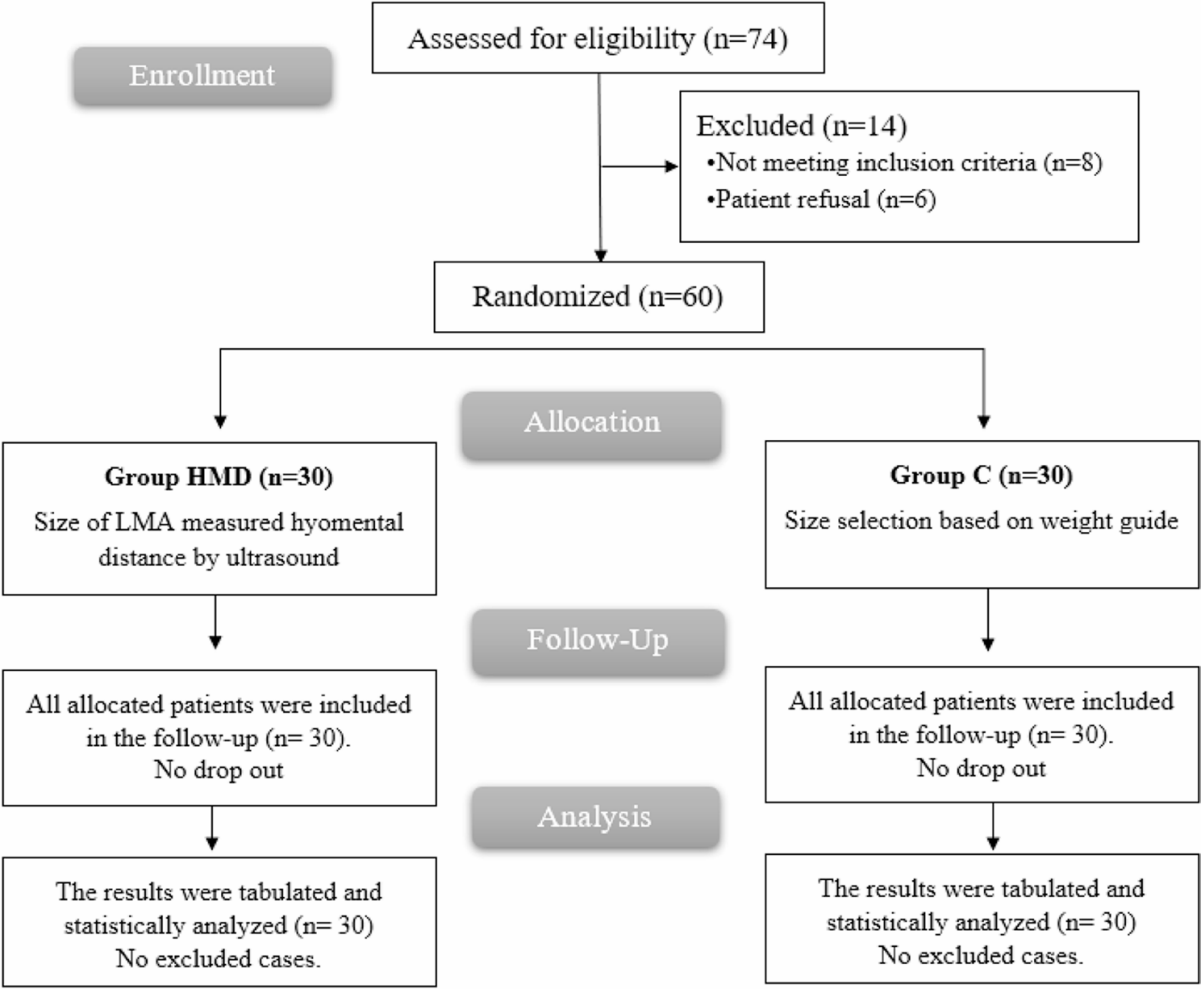
CONSORT flowchart of the enrolled patients

Seventy-four subjects were assessed for eligibility in this study; eight did not satisfy the requirements, six declined participation, and the remaining sixty patients were randomly allocated into two equal groups of thirty, with statistical analysis performed on all participants after their follow-up Fig. 1.

Demographic data was insignificantly different between both groups. Table 1.

**Table 1:** Demographic data of the studied groups

		Group HMD (*n* = 30)	Group C (*n* = 30)	*P* value	Mean difference, RR (95%CI)
Age (years) (min-max)		8.9 ± 2.99 (4–14)	7.73 ± 2.85 (3–12)	0.127	1.17(−0.34: 2.68)
Sex	Male	12 (40%)	16 (53.33%)	0.301	0.75(0.43:1.3)
	Female	18 (60%)	14 (46.67%)		
Weight (kg) (min-max)		34.87 ± 10.51 (17–49)	31.2 ± 11.55 (10–49)	0.204	3.67(−2.04: 9.37)
Height (cm)		133.85 ± 19.41	126.27 ± 18.54	0.127	7.58(−2.23: 17.39)
BMI (kg/m^2^)		19.51 ± 1.81	18.69 ± 2.65	0.168	0.3(−0.84: 1.44)
ASA physical status	I	20 (66.67%)	22 (73.33%)	0.573	0.91(0.65:1.27)
	II	10 (33.33%)	8 (26.67%)		

Data are presented as mean ± SD or frequency (%)

*RR* Relative risk, *BMI* Body mass index, *ASA* American Society of Anesthesiologists, *CI* Confidence interval

LMA size and HMD were insignificantly different between both groups. Table 2.

**Table 2:** LMA size and HMD of the studied groups

		Group HMD (*n* = 30)	Group C (*n* = 30)	*P* value	Mean difference, RR (95%CI)
LMA size	2	4 (13.33%)	7 (23.33%)	0.602	0.57(0.19:1.75)
	2.5	6 (20%)	5 (16.67%)		
	3	20 (66.67%)	18 (60%)		
HMD (cm)		3.52 ± 0.96	3.12 ± 0.95	0.113	0.4(−0.1: 0.89)

Data are presented as mean ± SD or frequency (%)

*RR* Relative risk, *CI* Confidence interval

The rate of successful intubation was significantly higher in the HMD group (93.33%, 28/30 cases) compared to the control group (70%, 21/30 cases) (*p* = 0.041), and an odds ratio of 1.33 (95% CI: 1.04–1.72). The mean of injury, the number of insertion attempts, and the mean OLP were insignificantly different between groups. The median five-point scale and total two-point score were significantly lower in Group HMD than in Group C (*P* < 0.05). Table 3.

**Table 3:** Successful intubation, injury rate, insertion attempts, OLP, (Five-point scale, and Two-point score) of the studied groups

	Group HMD (*n* = 30)	Group C (*n* = 30)	*P* value	Mean difference, RR (95%CI)
Successful intubation	28 (93.33%)	21 (70%)	0.041	1.33(1.04:1.72)
Injury rate	5 (16.67%)	7 (23.33%)	0.748	0.71(0.25:2)
Insertion attempts	1(1–2)	1.5(1–2.75)	0.631	0 (0–0)
OLP (cmH_2_O)	23.1 ± 4.29	24.07 ± 4.95	0.422	−0.97(−3.36: 1.43)
Five-point scale	1(1–2)	2(1–3)	0.036	0 (0–1)
Two-points score	0(0–1)	1(0–1.75)	0.018	0 (0–1)

Data are presented as median (IQR) or frequency (%)

*RR *Relative risk*, CI* Confidence interval, *OLP* Oropharyngeal leak pressure

The HMD can significantly predict successful intubation with 98% sensitivity, 72.7% specificity, 94.1% PPV, 88.9% NPV, and 93.3% accuracy. Weight-based criteria can significantly predict successful intubation with 87.8% sensitivity, 9.1% specificity, 81.1% PPV, 14.3% NPV, and 73.3% accuracy. The HMD can predict successful intubation better than weight-based (*P* < 0.001). Table 4.

**Table 4:** Role of weight-based criteria and HMD in the prediction of successful intubation

	Sensitivity	Specificity	PPV	NPV	Accuracy
Weight-based criteria	87.8%	9.1%	81.1%	14.3%	73.3%
HMD	98%	72.7%	94.1%	88.9%	93.3%

*PPV* Positive predictive value, *NPV* Negative predictive value

## Discussion

The appropriate selection of LMA size is crucial for ensuring optimal airway management and minimizing complications in pediatric anesthesia [1, 2]. Traditional weight-based criteria for LMA size selection may not always account for individual anatomical variations, leading to potential mismatches and suboptimal outcomes [4, 5, 8]. Recent research has explored alternative methods, such as US measurement of HMD, which may offer more precise and individualized approaches to selecting the size of LMA in pediatrics [10, 21, 22].

The US measurement of HMD may offer advantages over traditional weight-based criteria for pediatrics selection LMA size undergoing general anesthesia [10, 21]. The similar range of LMA sizes in both groups (*p* = 0.602), even though they used different selection methods (HMD-based vs. weight-based), shows that there are differences in anatomy even among the same weight categories. This emphasizes the importance of personalized sizing methods. This reinforces the need for individualized sizing approaches [5].

The mean HMD measurements showed no significant difference between groups (HMD: 3.52 ± 0.96 cm, C: 3.12 ± 0.95 cm, *p* = 0.113), suggesting HMD may be a reliable parameter across pediatric age groups [18]. Despite the differences in LMA size selection methods (HMD-based vs. weight-based), the consistency in HMD measurements between groups suggests that HMD may serve as a reliable anatomical parameter across pediatric age groups, independent of selection criteria [12].

The high predictive value of the HMD method aligns with previous research [13], extending its applicability to pediatric populations. Moreover, it reduces variability associated with weight-based criteria alone [15].

The HMD group demonstrated a markedly elevated intubation success rate (93.33%) compared to the C group (70%) (*p* = 0.041). Wang et al. [23] reported a correct LMA size selection rate of 77.14% using the HMD method, which, while lower than our success rate, still outperformed their weight-based group (51.51%, *p* = 0.027). This aligns with our findings, supporting the efficacy of HMD as a predictor of appropriate LMA size. Kim et al. [24] reported an 84% first-try LMA insertion success rate, which falls between our HMD and control group rates. However, their study primarily focused on US detection of LMA rotation rather than initial size selection, making direct comparisons challenging. Moreover, Petrisor et al. [13] and Khoury et al. [25] showed the effectiveness of US-based HMD for optimizing LMA placement in children.

While there was no statistically significant difference between the two groups, we did find that the HMD group had a lower injury incidence (16.67%) than the control group (23.33%). This trend suggests that the HMD method might contribute to safer LMA placement due to more accurate size selection. Wang et al. [23] reported similar findings, with injury rates of 14.29% in their HMD group and 21.21% in their weight-based group. The consistency strengthens the hypothesis that HMD-based LMA selection may reduce the risk of injury. This trend towards reduced injury rates in pediatric patients where minimizing airway trauma is crucial [1].

In our study, the median number of insertion attempts was similar, involving the HMD and control groups (1 and 1.5, respectively), with no statistical significance. This suggests that while the HMD method may improve success rates, the tries needed for successful insertion may not necessarily decrease. Wang et al. [23] reported identical results for insertion attempts (median 1, range 1–2) for both their HMD and weight-based groups.

Our study found comparable OLP values between the HMD group (23.1 ± 4.29 cmH_2_O) and the control group (24.07 ± 4.95 cmH_2_O), with no significant difference. Wang et al. [23] reported similar OLP values for both their HMD group and weight-based group (22 cmH_2_O, range 19–24 cmH_2_O), which closely aligns with our findings.

The HMD group performed better on the five-point scale (median 1 vs. 2, *p* = 0.036) and the two-point score (median 0 vs. 1, *p* = 0.018). These improved performance measures align with those of Koundal et al. [14], who demonstrated that the US at the point of care is a valuable tool for preoperative airway evaluation. Interestingly, none of the comparative studies [4, 22–24] utilized these specific scoring systems. This difference in assessment methods underscores the need for standardized evaluation criteria in future research to facilitate more direct comparisons between studies.

The HMD method showed higher sensitivity (98% vs. 87.8%), specificity (72.7% vs. 9.1%), PPV (94.1% vs. 81.1%), NPV (88.9% vs. 14.3%), and overall accuracy (93.3% vs. 73.3%, *p* < 0.001). Zheng et al. [22] reported varying predictive measures for different age groups using HMD in an extended position. For children aged 5–8 years, HMD < 3.9 cm showed a sensitivity of 0.88, specificity of 0.60, PPV of 0.10, NPV of 0.99, and accuracy of 0.61. For children aged 9–12 years, HMD < 4.2 cm demonstrated a sensitivity of 0.71, specificity of 0.83, PPV of 0.18, NPV of 0.98, and accuracy of 0.83. While these values differ from our results, they support the potential of HMD as a predictive tool, with variations possibly due to different age groups and specific measurement techniques.

Kim et al. [24] reported high predictive values for the US in detecting rotated LMAs, with sensitivity of 93%, specificity of 82%, PPV of 80%, NPV of 94%, and accuracy of 87%. While their focus was on LMA rotation rather than size selection, these results demonstrate the potential of US-based assessment in LMA management.

Its higher sensitivity indicates better identification of successful intubation cases, which is crucial in pediatric anesthesia for anticipating airway challenges [26]. The improved specificity suggests that the HMD method is far more effective at identifying potential intubation difficulties. This contrasts with the high false-positive rate of the weight-based criteria method, allowing for more targeted airway management strategies [11]. The HMD method showed better PPV and significantly higher NPV, indicating the HMD method’s superior reliability in identifying patients at risk of intubation challenges [27]. The overall accuracy was substantially higher for the HMD method, proving its superiority in predicting successful intubation in pediatric patients [10]. The potential of the HMD method to reduce airway-related complications is particularly relevant in optimizing airway management techniques for various pediatric surgical procedures [28].

This study is limited by its small sample size, single-center design, operator-dependent US measurements, lack of comparison with other predictive measurements and second-generation LMAs, exclusion of postoperative sore throat evaluation, a specific study population (subgroups) limiting broader applicability, and the fact that all LMAs were placed by one anesthesiologist, which may affect generalizability to less experienced providers.

Future studies should include a larger, more diverse sample across multiple centers to improve generalizability. Standardized training for US measurements can reduce operator dependence, and additional predictive measurements should be compared for a more comprehensive assessment. Evaluating second-generation LMAs and postoperative sore throat outcomes would provide a broader perspective. Including anesthesiologists with varying experience levels in LMA placement can enhance applicability to a wider range of providers.

## Conclusions

The US-measured HMD provides a superior method for selecting LMA size in children compared to traditional weight-based criteria. The HMD method showed significantly improved predictive value across all measures, including sensitivity, specificity, PPV, NPV, and overall accuracy. These improvements translated into higher rates of successful intubation, a trend towards reduced injury incidence, and better performance scores.

## Data Availability

Data is available on reasonable requests from corresponding author.

## Abbreviations

ASA: American Society of Anesthesiologists
HMD: Hyomental distance
IQR: Interquartile range
LMA: Laryngeal mask airway
NPV: Negative predictive value
PPV: Positive predictive value
US: Ultrasonography
